# Development of a theory-based instrument to identify barriers and levers to best hand hygiene practice among healthcare practitioners

**DOI:** 10.1186/1748-5908-8-111

**Published:** 2013-09-23

**Authors:** Judith Dyson, Rebecca Lawton, Cath Jackson, Francine Cheater

**Affiliations:** 1 Faculty of Health and Social Care, University of Hull, Cottingham Road, Hull, UK; 2 Department of Health Psychology, University of Leeds, Leeds, UK; 3 Bradford Institute for Health Research, Duckworth Lane, Bradford, UK; 4 Department of Health Sciences, University of York, Heslington, York, UK; 5 School of Nursing Sciences, Faculty of Medicine and Health Sciences, University of East Anglia, Norwich Research Park, Norwich, UK

**Keywords:** Evidence-based practice, Implementation, Hand hygiene, Instrument, Barriers and levers, Psychological theory, Compliance

## Abstract

**Background:**

A theoretical approach to assessing the barriers and levers to evidence-based practice (EBP) with subsequent tailoring of theoretically informed strategies to address these may go some way to positively influencing the delay in implementing research findings into practice. Hand hygiene is one such example of EBP, chosen for this study due to its importance in preventing death through healthcare associated infections (HCAI). The development of an instrument to assess barriers and levers to hand hygiene and to allow the subsequent tailoring of theoretically informed implementation strategies is reported here.

**Methods:**

A comprehensive list of barriers and levers to hand hygiene were categorised to the Theoretical Domains Framework (TDF) in a Delphi survey. These items formed the basis of an instrument that was tested to establish validity and reliability. The relationship between self-reported compliance with hand hygiene and barriers and levers to hand hygiene was also examined along with compliance according to where the barriers and levers fit within the domains of the TDF framework.

**Results:**

A 33-item instrument that tested well for internal consistency (α = 0.84) and construct validity (χ^2^/df = 1.9 [p < 0.01], RMSEA = 0.05 and CFA = 0.84) was developed. The relationship between self-reported compliance with hand hygiene moderately correlated with barriers identified by participants (total barrier score) (r = 0.41, n = 276, p <0.001). The greater the number of barriers reported, the lower the level of compliance. A one-way between groups multivariate analysis of variance was performed to investigate differences between those adopting high or low compliance with hand hygiene. Compliance was highest for this sample of participants among practitioners with high levels of motivation, strong beliefs about capabilities, when there were positive social influences, when hand hygiene was central to participants’ sense of professional identity and was easier to remember to do.

**Conclusions:**

This study has produced encouraging findings suggesting the potential for improved hand hygiene and resulting effects on the human and financial costs of healthcare associated infection. This study identifies a further potential use for the TDF.

## Background

Hand hygiene has been identified as the primary measure to reduce healthcare associated infection, which affects approximately 300,000 patients a year in England and costs the National Health Service over £1 billion [1]. However, compliance with hand hygiene practice is low despite national and internationally led strategies to improve this [2]. For these reasons and the fact that hand hygiene is a behaviour undertaken by all healthcare practitioners, it was chosen as the focus of the current study.

The delay in implementing research findings into practice is a well-documented problem [3]. Interventions to support implementation (*e.g*., interactive education, audit and feedback, or computerised reminders) are not consistently effective in leading to practice changes [4]. The development and/or selection of interventions to implement changes in practice often appears to be done on the basis of intuition rather than theory [5-7], and this has led to a call for a different approach. This approach involves: the accurate assessment of barriers and levers to implementation [4,8], and the subsequent tailoring of implementation strategies accordingly [4,9]; and a theoretical basis for this assessment of barriers and levers to change, and the tailored implementation interventions [10]. This proposed approach would also elucidate the mechanisms of change and begin to help us understand why some implementation strategies are more effective than others [11]. Behaviour change theory provides a sound theoretical basis for addressing issues of implementation [11].

There are many theoretical models explaining behaviour change [5,7,10], some of which have been applied to the implementation of evidence-based practice (EBP) [6,7,10]. The general conclusion from this work is that relevant psychological theories fit into three broad categories: those specific to the individual (*e.g*., cognitions, motivation, routine and learning style); the immediate social context (*e.g*., the influence of others, social norms and interactions); and the organisational context (*e.g*., culture and resources) [6]. The relevant theories are not distinct but often overlap, sometimes to a large extent [12].

The Theoretical Domains Framework (TDF) was developed by a group of health psychologists in the British Psychological Society [10] to provide a comprehensive overview of behaviour change theory based on 128 constructs from 33 theories of behaviour change and covering the individual, social and organisational context. Key constructs of behaviour change were grouped into 11 key domains: ‘knowledge,’ ‘skills,’ ‘social/professional role and identity,’ ‘beliefs about capabilities,’ ‘beliefs about consequences,’ ‘motivation and goals,’ ‘memory, attention and decision processes,’ ‘environmental context and resources,’ ‘social influences,’ ‘emotion’ and ‘behavioural regulation.’ A twelfth domain, ‘nature of behaviours’ was not included here. This domain is distinct from the others in that it is not a determinant of behaviour but rather a set of characteristics (*e.g*., frequent or one-off, approach or avoid) that can be used to describe the behaviour. Hand hygiene is an approach behaviour that should be performed frequently by healthcare practitioners, when they have patient contact. It is observable and constitutes a number of actions that need to be performed in a specific order.

A number of studies have used the TDF to elicit barriers and levers to implementation through interview [13-17]. Only one study was identified that had used the TDF as a basis for a theoretically-based instrument [18]. This could offer an expedient way of assessing barriers and levers in practice on a large scale. A review of existing tools to assess barriers to implementing EBP identified a few (for example, [19-21]), although none were explicitly underpinned by theory. To facilitate the use of theory in implementation research and practice, a tool is needed to enable researchers and practitioners to prospectively measure the theoretical determinants that represent barriers and levers to practice change. This knowledge can then be used to design appropriate theory-informed strategies to support change. In this paper, we present the development and testing of an instrument to understand the barriers and levers to hand hygiene, based on the TDF [10].

## Methods

### Instrument development

There were four stages of instrument development: a qualitative study to identify the barriers and levers to hand hygiene, a Delphi survey to categorise these barriers and levers according to the domains of the TDF, a design stage where items were selected for inclusion on the instrument, and a pilot study to ensure that the instrument was acceptable and comprehensible. Ethical approval was obtained from the National Research Ethics Service (08/H1306/31), the School of Healthcare Research Ethics Committee (SHREC/RP/132), and Research Governance approval was obtained from the NHS trusts involved in the study.

### Qualitative study: identifying the barriers and levers to hand hygiene

To ensure a comprehensive list of barriers and levers, a qualitative study was carried out with a sample of 70 healthcare practitioners (consisting of a wide spectrum of workers including doctors, nurses, therapists, porters and ward clerks) in three hospital sites. The results of this study are reported elsewhere [13]. From the qualitative study, 100 barriers and levers to hand hygiene practice were identified, which were compiled into statements for use in Step 2; for example, where a respondent said that confidence in their ability was a lever to hand hygiene, the statement produced was ‘I am confident in my ability to carry out hand hygiene.’

### A Delphi survey: categorising the barriers and levers according to the domains of the TDF

A two-round Delphi survey was carried out to assess the fit of the 100 barriers and levers to hand hygiene to each of the domains of the TDF. In total, 21 experts [22] in the fields of infection prevention and health psychology were asked to assess for fit each of the 100 barriers and levers to hand hygiene to the 11 domains of the TDF. This was achieved for 99 of the 100 barriers and levers at a level of 70% agreement or greater. The exception was the item ‘the hand hygiene guidelines are too long.’ This item was discarded.

### Selecting items for inclusion and designing the instrument

It is advised that, in the early stages of instrument development, the researcher should be as inclusive as possible, as poor items can be detected and removed during the subsequent process of testing [23,24]. The 99 barriers and levers identified through the qualitative study and categorised to the TDF domains in the Delphi survey were all considered for inclusion as items for the instrument. They were then removed where there was overlap. For example, ‘there are not enough sinks for hand hygiene’ was considered to overlap with ‘facilities are inadequate for hand hygiene.’ The initial 99 items became 81 after this process. These items are listed in Additional file 1 according to the domain of the TDF to which they were categorised through the Delphi survey. There were between 4 and 14 items in each domain.

Items were listed in random order in the instrument, and a 7-point Likert scale format was used. Participants were asked to circle the number that best reflected their opinion, ranging from 1 (strongly agree) to 7 (strongly disagree). At this stage, it recognised that although the questions in the category of ‘knowledge’ asked about sources of knowledge and beliefs about the effectiveness of hand hygiene in preventing infections, they did not directly test knowledge. It was therefore decided to add questions that tested knowledge. A review of the literature identified questions developed by the Institute for Healthcare Improvement [25] for use in monitoring improvement of hand hygiene in healthcare workers. These were added to the instrument at this stage and from this point forward are referred to as ‘knowledge testing questions.’

Care was taken to ensure the instrument was written in plain English and jargon was avoided. To avoid social desirability bias (participants responding favourably), participation was anonymous, and acquiescence bias (agreement without thought or if in doubt) was avoided by mixing questions so that when participants circled a number ranging from 1 to 7, this sometimes identified a barrier and sometimes a lever. Information and instruction for completion and return were provided on the instrument.

### Pilot study

A small pilot study was conducted with a sample of ten participants from one of the study hospital trusts who were identified by ward or departmental managers (study sites are described in detail below). Pilot participants were asked to complete the instrument and comment on the clarity of the items and suggest improvements that could be made. Pilot results demonstrated that the instrument was comprehensible, and no changes were made at this stage.

### Instrument testing

There were three stages of instrument testing that took place between June 2008 and September 2010. First, the instrument was tested for face validity, variability of response, skew and internal consistency within domains. Second, internal consistency within domains and construct validity was tested. Third, test-retest assessment was carried out.

Study sites were four hospital trusts in the North of England, reflecting diversity in terms of MRSA (Methicillin-resistant Staphylococcus aureus) rates and hygiene ratings. Participants were selected from a wide range of hospital areas (for example, accident and emergency departments, outpatient departments, wards) and from a mixture of occupational groups (for example, nurses, doctors, porters, physiotherapists). Recruitment took place through the charge nurses and heads of hospital departments.

### Face validity, variability of response, skew and internal consistency within domains

The purpose of the first round of testing was to perform preliminary tests to identify items most likely to provide valid measures of the 11 domains of the TDF, that is, those which demonstrated variability of response, good internal consistency, and relatively normal distributions. Based on estimates of response rates for postal questionnaires being approximately 33% [26] and in order to ensure a minimum of 50 returned instruments [27], 150 were distributed, again, via ward and departmental managers. Items were removed according to the principles of face validity (based on analysis of feedback from participants who were asked to offer criticism), a skew of greater than 3 in either direction [28], a lack of variability, defined as a standard deviation of less than 1.5 (this cut off point was set quite low as there were only 7 options on the Likert scale). Items were also removed in order to achieve a Cronbach’s alpha of 0.7 or more per domain, which was considered to demonstrate good correlation between items within the domain [29].

#### Analysis

Data were entered into SPSS v. 17. Descriptive statistics (frequencies) were used to summarise participants’ role and area of work (hospital department). Data were considered for variability of response, internal consistency, and skewness.

#### Results

A total of 56 participants (37.4%) responded from a range of hospital departments and included 40 nurses, 3 doctors, 2 porters, 2 radiographers, and a number of practitioners from other groups such as therapists, pharmacists and administrators. Areas of work included accident and emergency, surgical and medical wards, outpatients, paediatric wards and intensive care units.

Through this process, items were reduced from 81 to 68 with a minimum of 5 items per domain.

### Internal consistency within domains, construct validity, the relationship between barriers and levers, and compliance with hand hygiene and test-retest reliability

The purpose of the second round of instrument testing was construct validation, to see if the specific items of the instrument fitted within the domains of the TDF. Items numbering 68 suggests that a minimum sample of 340 respondents was required [27]. A total of 900 instruments were distributed by post via ward and departmental managers in order to achieve a minimum sample size (based on the 37% response rate achieved in round one). In order to demonstrate the potential utility of tailoring implementation interventions according to assessed barriers and levers to hand hygiene, the relationship between self-reported compliance with hand hygiene and barriers/levers to practice was investigated during this second round of instrument testing. As this second stage involved the most participants, these data were also used to examine the relationship between barriers and levers and compliance with hand hygiene.

#### Analysis

Data were entered onto SPSS v. 17. Descriptive statistics (frequencies) were used to summarise participants’ roles and areas of work (hospital department). Further analysis followed the steps listed below.

#### Internal consistency

Cronbach’s alpha was used and items were removed as necessary to achieve an alpha coefficient of 0.7 or above within domains.

#### Construct validity

**Figure 1:**
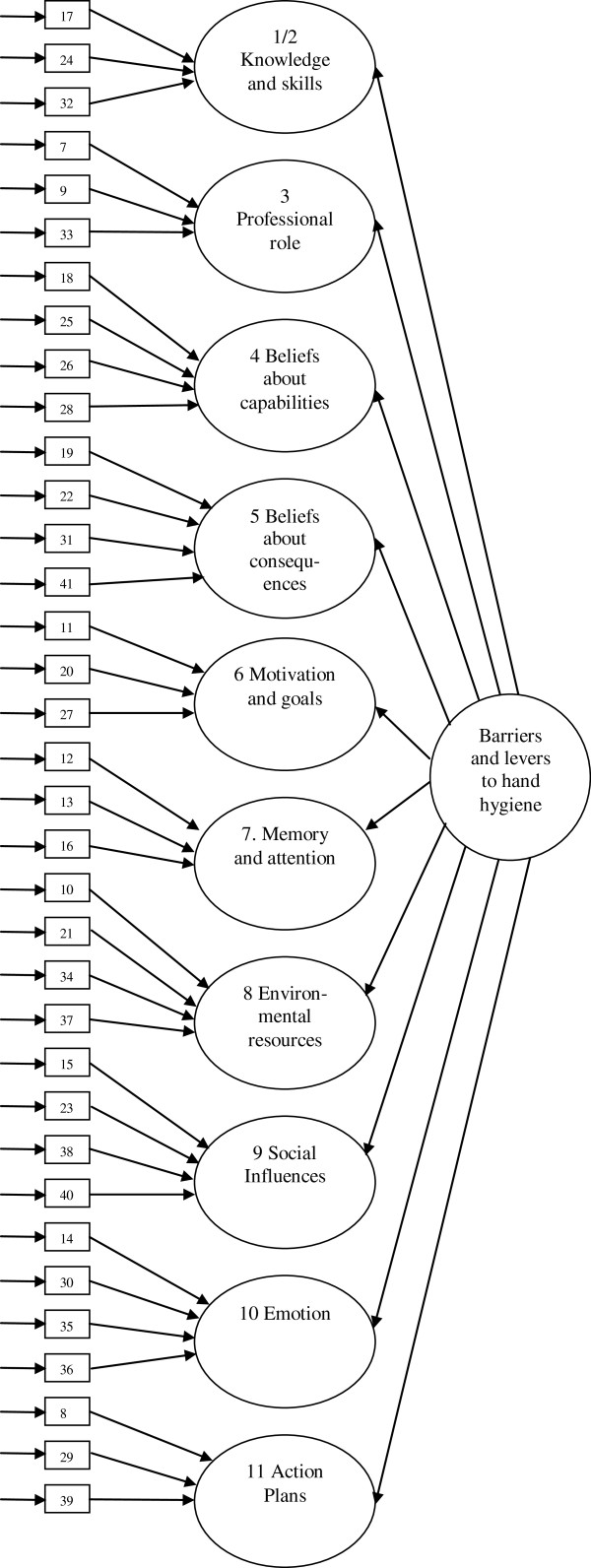
Factor structure of the BALHHI.

Confirmatory Factor Analysis (CFA) was performed using AMOS v. 17. to test whether the data from the individual items on the instrument fitted within the domains to which they had been allocated during the Delphi survey. Only variables with a skew greater than 3 and kurtosis index greater than 10 were of concern [28]. As no items exceeded these values, all were retained. A model was specified in AMOS v. 17 and tested for goodness of fit using three indices to measure absolute fit, parsimony, and comparative fit [30]: absolute fit based on Chi square to degrees of freedom ratio (χ^2^/df) of less than 2 [31]; parsimony of fit based on Root Mean Square Error of Approximation (RMSEA) of close to or less than 0.06 [30]; and comparative fit (CFA), based on an index ‘close to’ 0.95 [30]. The model was revised and the fit was retested. This was repeated until the model was judged to fit well according to the three measures of fit described above. Figure 1 demonstrates the final structure of The Barriers and Levers to Hand Hygiene Instrument (BALHHI). Squares represent items (numbers correspond with item numbers on the final instrument), the oval shapes represent the domains of the TDF, the first order latent variables, and the circle represents the overarching second order latent variable.

#### Knowledge testing questions

These were treated separately from the rest of the data and examined for face validity and variability of response.

### The relationship between barriers and levers and compliance with hand hygiene

Self-reported compliance with hand hygiene (measured as percentage) and total barrier score (1 = lever and 7 = barrier) was investigated using Pearson correlation coefficient. A one-way between groups multivariate analysis of variance was performed to investigate differences between those adopting high or low compliance with hand hygiene (based on a median split). The dependent variables were scores on the 10 domains of the TDF.

#### Results

In total, 354 participants (35.4%) returned completed instruments. The respondents included 201 nurses, 33 doctors, 9 porters, and a number of practitioners from other groups such as therapists, radiographers, and domestic staff. Areas of work included accident and emergency, surgical and medical wards, outpatients, paediatric wards, and intensive care units.

#### Internal consistency

The overall reliability for the instrument was 0.84. Seven items were removed because they reduced the alpha within their respective domains. An alpha of close to 0.7 was achieved for all domains except ‘beliefs about capabilities’ (α = 0.49). The items remaining after this process and the domains within which they fit formed the model for the confirmatory factor analysis carried out.

#### Construct validity

By the end of the process outlined in the stages above, the final model consisted of 33 items within 10 domains, and the fit was good; χ^2^/df = 1.9 (p <0.01), RMSEA = 0.05 and CFA = 0.84.

From the seven questions that had been added to test knowledge, two questions, ‘which of the following bacteria readily survive in the environment of the patient for days to weeks?’ and ‘which of the following statements about alcohol-based hand hygiene products is accurate?’ were incorrectly answered by the majority of participants (only 6% and 22% correct responses respectively), demonstrating poor variability of response. These were therefore removed, leaving five questions testing knowledge.

### The relationship between barriers and levers and compliance with hand hygiene

There was a medium negative correlation between self-reported compliance and total barrier score (r = −0.41, n = 276, p <0.001). That is, the greater the number of barriers, the lower the level of compliance for hand hygiene. The results of multivariate analyses are listed below according to domain. Compliance was highest (for this sample of participants) among people with high levels of motivation, strong beliefs about capabilities, when there were positive social influences, when hand hygiene was central to participant’s sense of professional identity and was easier to remember to do (all large effects, see Table 1).

**Table 1:** Relationship between self-reported compliance with hand hygiene and barriers according the domains of the TDF

**Domain**	**F (df)**	**Partial eta squared (effect)**
Knowledge and skills	4.39 (6, 324), p <0.001	0.075 (moderate)
Professional/social identity	9.8 (8, 321), p <0.001	0.196 (large)
Beliefs about capabilities	16.69 (6, 325), p <0.001	0.236 (large)
Beliefs about consequences	6.62 (7, 319) p <0.001	0.127 (large)
Motivation	15.84 (7, 309) p <0.001	0.264 (large)
Memory	8.9 (6, 329) p <0.001	0.14 (large)
Environment	4.13 (9, 300) p <0.001	0.111 (moderate)
Social Influences	5.83 (10, 310) p <0.001	0.158 (large)
Emotion	8.09 (5, 326) p <0.001	0.11 (moderate)
Action Planning	4.08 (6, 307) p <0.001	0.074 (moderate)

#### Test retest assessment

The purpose of the third and final round of instrument development was to assess the test-retest reliability, assessed by administering the instrument to the same sample of participants on two different occasions and calculating the correlation between the two sets of responses obtained [31]. If the phenomenon being measured is unchanged between time periods, the instrument is reliable [24,32]. The time between administering the two instruments is generally between 2 and 14 days, as this is not so long that things may have changed but not so short that participants remember what they answered on the first occasion rather than answering the question objectively [24]. However, due to the shift patterns of hospital workers and the postal time delay (internal university and hospital post as well as the external post service to send out and receive returned instruments), it was decided to separate the two occasions by one calendar month.

A total of 150 instruments were distributed (based on previous response rates) to achieve 50 returned instruments [27]. Reminders were sent to encourage return of the re-test instrument. The ‘Test’ instruments were distributed via ward and departmental managers, and on this occasion, participants were asked to provide contact details so that the ‘re-test’ instruments could be distributed. After omitting items after round two of testing, there were 33 items, and 5 knowledge test questions remained for round three (Additional file 2: Final Instrument). Communication with the infection prevention teams within each trust established that no changes in hand hygiene promotion were planned during the test-retest period. If individual work circumstances changed (another potential confounder), additional questions were included at the end of the retest to establish whether the change might have had an effect on the results.

#### Analysis

Descriptive statistics (frequencies) were used to summarise participants’ roles and areas of work (hospital department). Pearson coefficient was used to assess test retest reliability.

#### Results

A total of 69 participants (34.5%) returned the instrument on the first occasion. Of these, 50 (25%) returned the instrument on the second occasion. The respondents included 35 nurses, 2 doctors, 2 therapists, and a number of practitioners from other groups such as radiographers and domestic staff. Areas of work included accident and emergency, surgical and medical wards, outpatients, paediatric wards, and intensive care units. Pearson’s coefficient was calculated for the agreement between each item for the two time periods. All results were based on n = 50 and p *<*0.01. Pearson’s coefficient was greater than 0.3 for all items. Two items fell in the ‘medium correlation’ range of 0.30 to 0.49 (‘hand hygiene is a non-negotiable part of my role’ and ‘I am confident in my ability to carry out hand hygiene’), and the remaining 31 items fell in the ‘good correlation’ range of 0.5 or above. Following this, the Pearson’s correlation was calculated for the agreement between domains, all results were based on n = 50 and p <0.01, and results fell within the range of 0.5 or above ‘good correlation.’ The final instrument is illustrated in Additional file 2.

## Discussion

Our aim was to develop and test a theory-based diagnostic instrument, The Barriers and Levers to Hand Hygiene Instrument (BALHHI), to accurately and prospectively assess the barriers and levers to hand hygiene practice to inform subsequent tailoring of implementation strategies in order to improve practice. This was achieved, and to our knowledge, this is the first questionnaire to measure barriers and levers to hand hygiene that is informed by psychological theory. The implications of this for both research and practice are discussed below.

The following limitations are acknowledged. The TDF domain ‘skills’ had only three barriers or levers assigned to it during the survey. It was considered that knowledge and skills and related training were likely to overlap with regard to hand hygiene. As a result, the two domains were combined. The resulting domain contained three items: ‘there are adverts or newsletters about hand hygiene in my workplace,’ ‘hand hygiene training is available to me,’ and ‘hand hygiene guidelines are easily accessible.’ It is questionable whether these items actually reflect the absence or presence of skills, as all three refer predominantly to information and knowledge (although it is expected that any training programme relating to hand hygiene would include a skills component). When completing the instrument, practitioners generally did not identify knowledge or skills barriers. However, the knowledge test questions that were included in the instrument demonstrated that there were deficits in knowledge. This could be due to hand hygiene being perceived as ‘easy’ by healthcare practitioners. Hand hygiene may be viewed as a practice that does not require extensive knowledge, as it is not a practice carried out only by healthcare practitioners but also by members of the public. Similar findings were reported in a study of General Practitioners’ knowledge of delivering the Human Papiloma Virus Vaccination programme [17]. Practitioners reported their knowledge to be ‘high’ but when tested directly was ‘moderate.’ This questions the value of including items in an instrument that ask the practitioner about their knowledge rather than simply testing knowledge.

A further limitation is the possible lack of representativeness of the sample composition in terms of occupational role. The first and third stages of testing included only small numbers of participants (n = 56 and n = 50 respectively), and therefore not all occupational groups were included. Across the three stages of testing, participants from a range of occupational groups that come into direct contact with patients took part in the study; however, there were only small numbers of ancilliary staff (*e.g*., porters and domestic staff). Further research with more participants from these groups is recommended to ensure that the BALHHI is reliable and valid across different occupational groups. Finally, this study relied upon self-reported compliance with hand hygiene. It is recognised that direct observation of healthcare practitioners during patient care activity by a trained and validated observer is the gold standard for hand hygiene monitoring [2]. Further research, whereby the BALHHI is tested against observed behaviour is needed.

The development of this instrument identifies a number of implications for research and practice. A limited number of instruments that pertain to the assessment of barriers and levers to evidence-based practice (EBP) have been developed, but no instrument was identified that assessed the barriers and levers to hand hygiene. While previously published instruments have been useful in identifying the barriers and levers to EBP, their main limitation is that they have no explicit underlying theoretical basis and have not been tested in tailoring implementation strategies. The instrument developed here is based on a theoretical framework, which allows the subsequent tailoring of implementation strategies. The main implication of this is the possibility of moving from the prospective assessment of barriers and levers to subsequent tailoring to theoretically informed implementation strategies. Abraham and Michie recently carried out a review of the relevant literature and identified a set of distinct, theory-linked definitions of behaviour change techniques [33]. This was developed further, and behaviour change techniques were linked to the theoretical constructs forming the 11 domains of the TDF [34]. That is, each technique was considered as to whether or not it would be effective as part of an intervention to assess behaviour with respect to each of the domains. Having assessed barriers and levers to hand hygiene using the instrument developed and reported here, it should be a straightforward process to select the appropriate behaviour change strategy or strategies (according to the work of Michie *et al*. [34]) to these barriers and levers according to the domain within which they fit. The work carried out here demonstrates a clear link between the barriers and levers to hand hygiene and self-reported hand hygiene compliance, which supports the viability of such an approach. In this study, we found ‘social influences,’ ‘environmental context and resources,’ and ‘memory attention and decision processes’ to be the top barriers/absence of levers to hand hygiene. However, this varied according to occupational group. For example, for porters this was ‘memory attention and decisions processes’; for doctors ‘social influences.’ The greatest barriers/absence of levers for practitioners working in accident and emergency care was ‘environmental’; for practitioners working in the intensive care this was ‘social influence.’ This knowledge supports the need to tailor interventions according to assessed barriers and levers, and from this could be used to inform the design of an intervention to target these determinants.

The TDF has now been used within a range of implementation behaviours, different clinical settings, and in a number of different ways [35,36]. This study has demonstrated the potential of the TDF to be adapted to an instrument format that would provide a means of surveying large numbers of healthcare practitioners.

There are potential benefits to practitioners of using a questionnaire rather than an interview approach when using the TDF; for example, this allows the inclusion of larger numbers of practitioners and is less time consuming. As such, this approach would also potentially allow the comparison of barriers and levers according to different occupational groups and areas of work. However, further research questions remain. For example, is this approach feasible to use in real world practice? Can interventions be tailored according to the barriers and levers identified using this instrument? How many domains should be focused on when selecting interventions? Can behaviour change strategies be adapted pragmatically for use within clinical settings with healthcare practitioners? The instrument developed and reported here was tested for validity and reliability. The next step is to investigate whether such an instrument can be used as the basis for tailoring implementation strategies according to the identified barriers and levers. The results presented here do demonstrate that the barriers and levers identified by the practitioners completing the instrument relate to their own hand hygiene compliance. This suggests that targeting these for intervention is likely to result in improved hand hygiene behaviour.

Our next step is to adapt the BALHHI from a paper-based instrument into an interactive software package. This package will be designed to present practitioners with theoretically informed strategies designed to improve hand hygiene. These strategies will be tailored according to the domains within which the assessed barriers and levers to practice fall and will be evaluated to see if improvements in hand hygiene result.

## Conclusions

The instrument reported here was designed to allow tailoring of theoretically informed implementation strategies based on the initial assessment of barriers and levers to hand hygiene. The potential implications of this are improved hand hygiene practice and the resulting positive effects on the human and financial costs of healthcare associated infection.

However, although the instrument showed good levels of validity and reliability, it now requires testing for its effectiveness in the tailoring of implementation strategies and the subsequent effects on hand hygiene compliance in a randomised controlled trial.

### Consent

Participants were informed of the intentions to publish the findings of this study and gave consent through the completion and returning of the questionnaire sent to them.

## Abbreviations

BALHHI: Barriers and levers to hand hygiene instrument; CFA: Confirmatory factor analysis; EBP: Evidence-based practice; HCAI: Healthcare associated infection; MRSA: Methicillin-resistant *Staphylococcus aureus*; TDF: Theoretical domains framework.

## Competing interests

The authors declare they have no competing interests.

## Authors’ contributions

JD managed the day-to-day running of this study with the supervision and substantial contribution of CJ, RL and FC. JD wrote the first manuscript of this paper, and the authors listed commented on and added to all drafts. All authors read and approved the final manuscript.

## Supplementary Material

Additional file 1 Items linked to the TDF. Click here for file

Additional file 2 Final Instrument. Click here for file
