# Supplementary material for: Development of a theory-based instrument to identify barriers and levers to best hand hygiene practice among healthcare practitioners
Source: Implement Sci. 2013 Sep 23;8:111. doi: 10.1186/1748-5908-8-111 (PMC3850814; doi:10.1186/1748-5908-8-111)
Supplement: Additional file 1 — Items linked to the TDF. [file 1748-5908-8-111-S1.pdf]

Additional File 1: Items linked to the TDF

Psychological Domain (abbreviated)	Barrier or lever to hand hygiene (HH)
1 Knowledge	E learning about HH<br>Don't believe that HH reduces infections in hospitals<br>Information for patients improves their HH<br>Adverts relating to HH inform practice<br>A HH newsletter helps improve practice<br>Advice from occupational health regarding sore hands improves HH<br>Basic training does not cover HH sufficiently<br>Training lag – staff are insufficiently trained for specialist posts<br>Available HH guidelines
2 Skills	Not computer literate<br>**HH training is tailored to professional group and need** appears also in professional role<br>Good HH training helps
3 Professional Role	HH is embedded into professional practice<br>HH is a non negotiable part of the job<br>Some professional groups are more likely to clean hands than others<br>Respect for patient improves HH<br>Board to ward responsibility improves practice<br>**HH training is tailored to professional group and need** appears also in skills<br>HH is better when the practitioner takes pride in their work<br>**Professional culture influences HH** appears also in professional role
4 Beliefs about Capabilities	A confidence in HH ability aids good practice<br>Full hands e.g. carrying equipment makes HH difficult<br>It is impossible to do HH as frequently as needed<br>Afraid to ask others to carry out HH
5 Beliefs about Consequences	Sore hands reduces HH<br>Patient may catch an infection and die if HH omitted<br>HH carried out to prevent and infection to self<br>Fear of disciplinary action of HH not carried out<br>HH carried out to prevent complaint from patient<br>The thought of cost of infections in hospital improves HH<br>HH creates patient confidence<br>**Criticised when HH missed** appears also in social influences<br>**Good attitude promotes HH** appears also in motivation and goals<br>HH avoids getting a bad reputation<br>Name and shame – being made an example of if HH is omitted<br>Challenged when HH not carried out<br>There is zero tolerance to poor HH<br>A certificate for good HH<br>Using the "glow and show" light box identifies unclean hands and influences HH<br>The organisation would lose status if HH not carried out<br>Don't want to feel responsible for infecting patient so carry out HH<br>Blame from others if HH not carried out

		Self-blame if HH omitted
6	Motivation and goals	More important things to do than HH<br>Enthusiastic about HH<br>Good intentions result in better HH<br>**Good attitude promotes HH** appears also in consequences<br>Belief that HH is not important<br>Complacent about HH<br>Emergencies and other priorities prevent HH<br>HH is a priority<br>Disagree with HH guidelines<br>Can't be bothered to carry out HH
7	Memory and attention	Forgets HH<br>It is automatic to clean hands<br>**Infection promotion notice boards prompt HH** appears also in environmental resources<br>HH is a good habit<br>Forget HH when tired<br>Infection promotion notice boards inform HH<br>Adverts relating to HH prompt practice
8	Environmental resources	Type of ward environment<br>Necessary equipment is too expensive<br>There are not enough sinks for good HH<br>Facilities are inadequate for HH<br>Some government policies make HH difficult (e.g. bed occupancy)<br>Hand cream encourages HH<br>Gel is always available<br>A cluttered environment prohibits HH<br>Inefficient systems of care inhibit HH<br>**Infection promotion notice boards prompt HH** appears also in memory and attention<br>Staff/skill mix is important if HH is to be carried out<br>No time to attend HH courses<br>Working in more than one area makes HH difficult (e.g. hospital and community based)<br>Poor staffing levels make HH difficult
9	Social influences	Strong leadership makes HH more likely<br>Reluctant to let the team down by omitting HH<br>Positive ward culture increases the likelihood of HH<br>The influence of peer pressure leads to better HH<br>HH champions help with HH compliance<br>Patients expect good HH from staff<br>Supervision from seniors improves HH<br>Opinion leaders promote HH<br>**Criticised when HH missed** appears also in consequences<br>Praise makes HH more likely<br>The reintroduction of Matrons improves HH<br>Infection prevention and control teams influence HH<br>A practitioner (link) is responsible for communicating with the infection prevention team<br>Good role models improve HH

	Encouragement helps HH compliance<br>Nobody else bothers with HH<br>**Professional culture influences HH** appears also in professional role<br>Reluctant to prompt senior staff with HH<br>Managers and the IPC team are very motivating with HH
10 Emotion	Fear of ward closure due to infection if HH omitted<br>Anticipated guilt or regret if hands are not cleaned<br>Feel guilty if HH omitted<br>Feel ashamed if HH omitted<br>Frustrated when others omit HH<br>Embarrassed to fail HH audit<br>Angry if HH not carried out
11 Action Plans	Several improvement strategies at the same time make HH more likely<br>Government and organisational targets mean HH is more likely<br>Audit and feedback improves HH
