# Supplementary material for: Development of a theory-based instrument to identify barriers and levers to best hand hygiene practice among healthcare practitioners
Source: Implement Sci. 2013 Sep 23;8:111. doi: 10.1186/1748-5908-8-111 (PMC3850814; doi:10.1186/1748-5908-8-111)
Supplement: Additional file 2 — Final Instrument. [file 1748-5908-8-111-S2.pdf]

Additional File 2: Final Instrument

Barriers and Levers to Hand Hygiene Instrument (BALHHI)

Information and Instructions

- This questionnaire has been developed because research tells us that hand hygiene will vary from hospital to hospital, between different wards and departments and also according to the role of different practitioners. We are trying to identify the factors that influence hand hygiene.
- The information you give will enable the identification of the improvement strategies that will be the most effective in supporting good hand hygiene.
- Simply consider each statement in the light of your own hand hygiene and circle the number that demonstrates to what extent you agree or disagree with the statements given.
- It is anticipated that this will take about 10 minutes.
- Your responses will be anonymous as you do not need to put your name on the questionnaire.

1. What is your job title? (e.g. staff nurse) .....………………………….....................

2. How long have you worked in health care? (In full years)

3. What area of the hospital do you work in? ....
   (e.g. elderly Care)

4. Are you male or female (please circle) M F

5. To what extent do you consider you usually comply with good practice guidelines for hand hygiene? (Times you clean your hands compared with opportunities to do so – 0 to 100%) ________ %

6. To what extent do you consider your colleagues in your department comply with good practice guidelines for hand hygiene? ________ %

Please consider your own hand hygiene. Then circle the number between 1 and 7 that best reflects your opinion at present.

	Strongly agree						Strongly disagree
7. I engage in hand hygiene out of respect for my patients	1	2	3	4	5	6	7
8. Government targets have led to improvements in my hand hygiene	1	2	3	4	5	6	7
9. Hand hygiene is a non-negotiable part of my role	1	2	3	4	5	6	7
10. It is difficult for me to attend hand hygiene courses due to time pressure	1	2	3	4	5	6	7
11. I feel complacent about hand hygiene	1	2	3	4	5	6	7
12. Sometimes I miss out hand hygiene simply because I forget it	1	2	3	4	5	6	7
13. Hand hygiene is not second nature for me	1	2	3	4	5	6	7
14. I feel angry if hand hygiene is not carried out by others	1	2	3	4	5	6	7
15. When staff engage in hand hygiene they are praised	1	2	3	4	5	6	7
16. I am more likely to forget hand hygiene if I am tired	1	2	3	4	5	6	7
17. Hand hygiene training is available to me	1	2	3	4	5	6	7
18. There are some practical barriers to hand hygiene because of my particular job/role	1	2	3	4	5	6	7
19. If I do not engage in hand hygiene I may catch an infection	1	2	3	4	5	6	7
20. I cannot be bothered with hand hygiene	1	2	3	4	5	6	7
21. Some government targets make hand hygiene more difficult (such as high bed occupancy)	1	2	3	4	5	6	7
22. If I omitted hand hygiene I would blame myself for infections	1	2	3	4	5	6	7
23. I engage in hand hygiene because I do not want to let the team down	1	2	3	4	5	6	7
24. There are adverts or newsletters about hand hygiene in my workplace	1	2	3	4	5	6	7
25. I am reluctant to ask others to engage in hand hygiene	1	2	3	4	5	6	7
26. The frequency of hand hygiene required makes it difficult for me to carry it out as often as necessary	1	2	3	4	5	6	7
27. I disagree with some parts of the hand hygiene guidelines	1	2	3	4	5	6	7

	Strongly agree						Strongly disagree
28. I am confident in my ability to carry out hand hygiene	1	2	3	4	5	6	7
29. Hospital targets relating to infection or hand hygiene has led to improvements in my hand hygiene	1	2	3	4	5	6	7
30. I feel frustrated when others omit hand hygiene	1	2	3	4	5	6	7
31. If I engage in hand hygiene it improves patient confidence	1	2	3	4	5	6	7
32. Hand hygiene guidelines are easily accessible	1	2	3	4	5	6	7
33. Hand hygiene is part of my professional culture	1	2	3	4	5	6	7
34. My environment is cluttered	1	2	3	4	5	6	7
35. I feel guilty if I omit hand hygiene	1	2	3	4	5	6	7
36. I feel ashamed if I omit hand hygiene	1	2	3	4	5	6	7
37. My area of work has poor staffing levels	1	2	3	4	5	6	7
38. Supervision from senior staff means that carrying out hand hygiene is easier for me	1	2	3	4	5	6	7
39. Some strategies designed to improve hand hygiene influence my practice	1	2	3	4	5	6	7
40. My hand hygiene is encouraged by others	1	2	3	4	5	6	7
41. If I miss out hand hygiene I will be subject to disciplinary action	1	2	3	4	5	6	7

42. In which of the following situations should hand hygiene be performed (circle as many letters as apply).

a. Before having direct contact with a patient
b. Before inserting an invasive device (e.g. catheter)
c. When moving from a contaminated body site to a clean body site during an episode of patient care
d. After having direct contact with a patient or with items in the immediate vicinity of the patient
e. After removing gloves

43. If your hands are not visibly soiled or visibly contaminated with blood or other material, which is most effective for reducing the number of disease causing bacteria? (circle one letter corresponding to the single best answer)

a. Washing hands with plain soap and water
b. Washing hands with an antimicrobial soap and water
c. Applying 1.5 to 3ml of alcohol-based hand rub to the hands and rubbing hands together until they feel dry

44. How are antibiotic-resistant bacteria most frequently spread from one patient to another in health care settings? (circle one letter corresponding to the single best answer)

a. Airborne spread resulting from patients coughing or sneezing
b. Patients coming in contact with contaminated equipment
c. From one patient to another via the contaminated hands of clinical staff
d. Poor environmental maintenance

45. Which of the following infections can be potentially transmitted from patients to clinical staff if appropriate glove use and hand hygiene are not performed? (Circle as many letters as apply)

a. Herpes simplex virus infection
b. Colonisation or infection with MRSA (methicillin-resistant Staphylococcus aureus)
c. RSV (Respiratory syncytial visus infection)
d. Hepititis B virus infection

46. Clostridium difficile (the cause of antibiotic-associated diarrhoea) is readily killed by alcohol based hand hygiene products. (Circle one letter corresponding to the single best answer)

a. True
b. False

Thank you for participating!
Please return this questionnaire to:

Questionnaire developed by Judith Dyson (j.dyson@hull.ac.uk), Francine Cheater, Cath Jackson and Rebecca Lawton
Questions 75 to 81 are adapted from: Institute for healthcare improvement, How to guide: A guide for improving practices among health care workers, 2009. http://www.shea-online.org/Assets/files/IHI_Hand_Hygiene.pdf
